# Bioactive Chemical Composition of Cannabis Extracts and Cannabinoid Receptors

**DOI:** 10.3390/molecules25153466

**Published:** 2020-07-30

**Authors:** Yi Yang, Rupali Vyawahare, Melissa Lewis-Bakker, Hance A. Clarke, Albert H. C. Wong, Lakshmi P. Kotra

**Affiliations:** 1 Department of Pharmaceutical Sciences, Leslie Dan Faculty of Pharmacy, University of Toronto, Toronto, ON M5S 3M2, Canada; William.Yang@uhnresearch.ca; 2 Centre for Molecular Design and Preformulations, and Krembil Research Institute, University Health Network, Toronto, ON M5G 1L7, Canada; rupali.vyawahare@uhnresearch.ca (R.V.); melissa.lewis@uhnresearch.ca (M.L.-B.); 3 Department of Anesthesia and Pain Management, Toronto General Hospital, University Health Network, Toronto, ON M5G 1L7, Canada; hance.clarke@uhn.ca; 4 Campbell Family Mental Health Research Institute, Centre for Addiction and Mental Health, and the Departments of Psychiatry and Pharmacology, Faculty of Medicine, University of Toronto, Toronto, ON M5T 1R8, Canada; albert.wong@utoronto.ca; 5 Multi-Organ Transplant Program, Toronto General Hospital, Toronto, ON M5G 1L7, Canada

**Keywords:** medical cannabis, tetrahydrocannabinol, cannabidiol, cannabinoid receptors, chemoinformatics, partial least squares analysis, quantitative structure-activity relationship

## Abstract

Cannabis is widely used as a therapeutic drug, especially by patients suffering from psychiatric and neurodegenerative diseases. However, the complex interplay between phytocannabinoids and their targets in the human receptome remains largely a mystery, and there have been few investigations into the relationship between the chemical composition of medical cannabis and the corresponding biological activity. In this study, we investigated 59 cannabis samples used by patients for medical reasons. The samples were subjected to extraction (microwave and supercritical carbon dioxide) and chemical analyses, and the resulting extracts were assayed in vitro using the CB_1_ and CB_2_ receptors. Using a partial least squares regression analysis, the chemical compositions of the extracts were then correlated to their corresponding cannabinoid receptor activities, thus generating predictive models that describe the receptor potency as a function of major phytocannabinoid content. Using the current dataset, meaningful models for CB_1_ and CB_2_ receptor agonism were obtained, and these reveal the insignificant relationships between the major phytocannabinoid content and receptor affinity for CB_1_ but good correlations between the two at CB_2_ receptors. These results also explain the anomalies between the receptor activities of pure phytocannabinoids and cannabis extracts. Furthermore, the models for CB_1_ and CB_2_ agonism in cannabis extracts predict the cannabinoid receptor activities of individual phytocannabinoids with reasonable accuracy. Here for the first time, we disclose a method to predict the relationship between the chemical composition, including phytocannabinoids, of cannabis extracts and cannabinoid receptor responses.

## 1. Introduction

**Figure 1:**
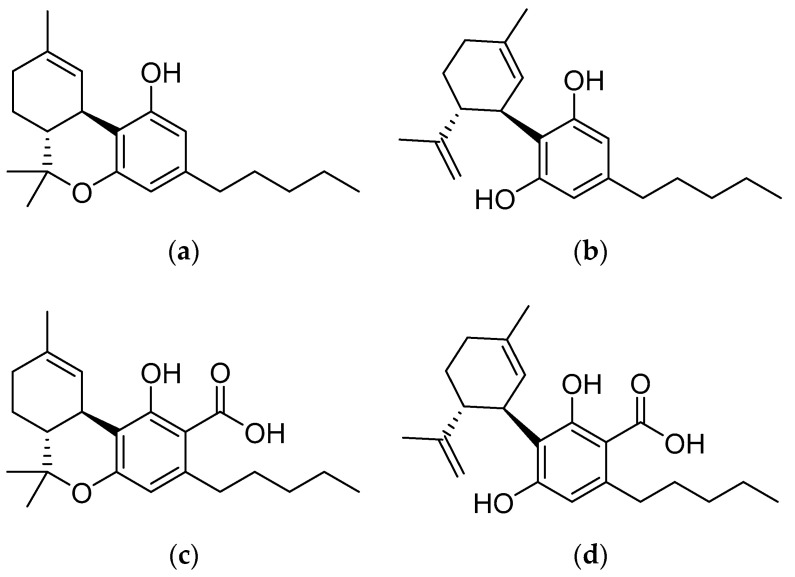
Major phytocannabinoids present in *C.* *sativa*: (**a**) *Δ^9^*-tetrahydrocannabinol, (**b**) cannabidiol, (**c**) *Δ^9^*-tetrahydrocannabinolic acid, and (**d**) cannabidiolic acid.

In recent years, cannabis has arguably become one of the most popular natural products across the globe. *Cannabis sativa*, a member of the herbaceous *Cannabaceae* family of plants, produces more than 568 unique compounds, of which more than 100 belong to the unique class of phytocannabinoids [1,2]. These are organic molecules with a polyphenolic structure. Phytocannabinoids such as *Δ^9^*-tetrahydrocannabinol (*Δ^9^*-THC), cannabidiol (CBD), *Δ^9^*-tetrahydrocannabinolic acid (*Δ^9^*-THCA), and cannabidiolic acid (CBDA) (Figure 1) exhibit a wide array of pharmacodynamic interactions in the human receptome [3]. Cannabinoids primarily interact with cannabinoid receptor types 1 and 2 (CB_1_ and CB_2_, respectively), but they can also interact with other receptors such as serotonin receptors and vanilloid-type transient receptor potential (TRPV) channels, which result in the physiological effects of cannabis, including psychoactivity or euphoria, motor impairment, analgesia, and immunomodulation [4,5,6].

The medicinal properties of cannabis and its derivative products make them potential treatments for managing the symptoms of several neurological and psychiatric disorders; they are also increasingly popular alternatives to opioids and are used by patients suffering from nausea, epilepsy, neuropathic pain, and post-traumatic stress disorder (PTSD) [7,8,9]. In most jurisdictions around the world, medical cannabis is accessible to patients, although it does not possess a drug identification number or a similar authorization by relevant drug control authorities [10,11]. There are hundreds of varieties of medical cannabis available in the market and these can be obtained in the forms of dried flowers, cannabis extracts, and infused oils [12,13]. The composition of phytocannabinoids in these cannabis varieties can vary significantly, leading to a range of biological and physiological responses; for example, the *Δ^9^*-THC content in cannabis samples can range from 0.14% to more than 25%, and, depending on the concentration of other phytocannabinoids, the resulting biological activities vary [12].

Interestingly, the variety (based on chemical composition), dose, and the route of administration of medical cannabis are largely determined by patients through self-titration with healthcare professionals playing an advisory role [7,11]. In fact, there have been no systematic studies investigating the relationship between the chemical constituents of different medical cannabis varieties and their corresponding combined therapeutic effects and receptor activities. Nevertheless, it is important to understand the differences between the natural product (which is a complex mixture of bioactive compounds) and individual bioactive compounds because the pharmacological responses produced by whole cannabis or cannabis extracts are different from those of single phytocannabinoids [13,14].

Here, through a series of experiments comprising chemical extractions, quantitative analyses, and CB_1_/CB_2_ receptor activity profiling, we have undertaken a comprehensive investigation of 59 cannabis samples, their chemical compositions, and receptor activities and identified if there are any relationships. In doing so, we hope to uncover any relationships between the chemical makeup and receptor activity of the medical cannabis samples. Such relationships will help explain differences in physiological effects caused by the different chemical compositions of cannabis samples. This may also lead to evidence of synergistic/antagonistic effects between the various phytocannabinoids present in medical cannabis and their relative concentrations.

## 2. Results

Fifty-nine samples of cannabis were collected from patients consuming cannabis for medical purposes. Among these samples, forty-five were dried cannabis samples, whereas the remaining fourteen samples were cannabis oils or resins. The concentrations of the four tested phytocannabinoids CBD, *Δ^9^*-THC, CBDA, and *Δ^9^*-THCA in all 59 cannabis samples varied significantly (Table 1 and Table 2). In our previous work, we showed that such variations can be a result of the different extraction methods employed, and, thus, extraction methods are important for quality control and quality assurance purposes [15]. Here, our focus is to investigate the effects of variations in phytocannabinoid concentrations on receptor responses and determine if there are quantitative correlations between the chemical composition and biological activities. In these cannabis extracts, the CBD, *Δ^9^*-THC, CBDA, and *Δ^9^*-THCA concentrations of a weight-by-weight (*w*/*w*) basis were 0–51.7%, 0–73.1%, 0–60.3%, and 0–76.1%, respectively (Table 1). Samples extracted using microwave assisted extraction (MAE) primarily contained decarboxylated phytocannabinoids, i.e., CBD and *Δ^9^*-THC, which was expected because of the high temperature produced using this extraction procedure, whereas samples obtained via supercritical fluid extraction (SFE) contained higher proportions of CBDA and *Δ^9^*-THCA. These differences contributed to a diverse sample set for the receptor activity evaluation. The cannabis oil samples, as received from various sources, are diluted solutions of phytocannabinoids; thus, the CBD, *Δ^9^*-THC, CBDA, and *Δ^9^*-THCA concentrations (*w*/*v*) varied from 0–8.9%, 0–4.6%, 0–31.7%, and 0–15.3%, respectively (Table 2). All but one of the cannabis oil samples contained only CBD and *Δ^9^*-THC.

The agonist and antagonist activities for all 59 cannabis samples, the pure cannabinoids (*Δ^9^*-THC, CBD, *Δ^9^*-THCA, and CBDA), and the reference compounds (CP55940, SR-141716, and AM-630) at CB_1_ and CB_2_ were evaluated, and the results are presented in Table 1 and Table 2. The receptor activities (half-maximal effective concentrations (EC_50_) or half-maximal inhibitory concentrations (IC_50_), for the characterization of agonist and antagonist effects, respectively) for each cannabis sample were calculated using the concentration of the phytocannabinoid present in the highest proportion (i.e., the major phytocannabinoid) in the corresponding cannabis sample. Notably the receptor activities of several extracts were poor or required concentrations of the major phytocannabinoids in excess of 10 µM, resulting in solubility issues and being physiologically impractical. These cannabis extracts were, thus, considered inactive.

Subsequently, we performed chemoinformatic analyses using partial least squares (PLS) methods, which are widely used in quantitative structure-activity relationship (QSAR) analyses to relate the phytocannabinoid concentrations and receptor responses. In a traditional QSAR, a dependent variable, such as receptor response, is correlated with physicochemical descriptors, such as octanol/water partition coefficients or Hammett constants derived for a set of molecules, to generate mathematical models. These models can explain the relevance of various molecular features to the observed biological activities and may enable the prediction of the physicochemical properties of untested compounds [16]. Using a similar strategy, we correlated the CB_1_ and CB_2_ receptor potencies of the medical cannabis samples to the four cannabinoid concentrations using PLS regression analysis to obtain QSAR-like prediction models (Table S2).

The concentrations of the four phytocannabinoids quantified in the 59 cannabis samples were fit against the corresponding agonist and antagonist activities of each cannabis sample at CB_1_ and CB_2_. Three subsets emerged with significant statistical power, containing sufficient data points for the generation of the corresponding mathematical models (Equations (1)–(3)).

The first relationship, modeled by Equation (1), is between the linear terms of the concentrations of CBD, *Δ^9^*-THC, and CBDA, and the quadratic term of the *Δ^9^*-THCA concentration, describing the corresponding agonist activities at the CB_2_ receptor (EC_50_):

Log(EC_50_) = −2.967 CBD − 1.649 × 10^−2^*Δ^9^*-THC + 5.143 × 10^−3^ CBDA + 1.363 × 10^−4^*Δ^9^*-THCA^2^ + 3.765 (1)

where *n* = 15, *r*^2^ = 0.842, *Q*^2^ = 0.784, RMSE = 0.338, Components = 1. The second relationship between the phytocannabinoid composition of the cannabis oil samples and their CB_1_ receptor agonism is described by Equation (2):

Log(EC_50_) = 0.142 CBD + 5.089 × 10^−2^*Δ^9^*-THC^2^ − 2.356 × 10^−2^ CBDA − 3.833 × 10^−2^*Δ^9^*-THCA + 1.510 (2)

where *n* = 9, *r*^2^ = 0.679, *Q*^2^ = 0.304, RMSE = 0.260, Components = 3. The third meaningful relationship between the phytocannabinoid composition of the cannabis extracts and their CB_1_ receptor potencies as agonists is described by Equation (3):

Log(EC_50_) = 5.479 × 10^−3^ CBD − 6.151 × 10^−3^*Δ^9^*-THC + 1.085 × 10^−4^ CBDA^2^ + 5.882 × 10^−3^*Δ^9^*-THCA + 2.681 (3)

where *n* = 42, *r*^2^ = 0.204, *Q*^2^ = 0.140, RMSE = 0.598, Components = 1. 

**Figure 2:**
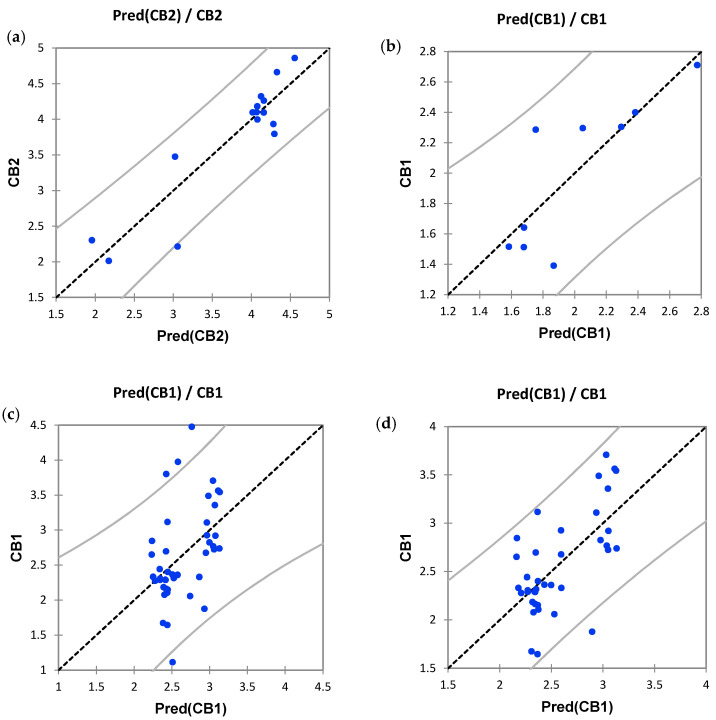
Distribution of calculated versus experimental receptor potencies for the models described by Equations (1)–(3) (above) (**a**–**c**, respectively) and Equation (4) (vide infra, **d**).

Figure 2 illustrates the distributions of the experimental and predicted receptor potencies for the three models (Equations (1)–(3) and Figure 2a–c, respectively).

## 3. Discussion

Individual phytocannabinoids exert different physiological effects on the human endocannabinoid system and other receptors, albeit with different potencies at each receptor. For example, *Δ^9^*-THC acts as a potent partial agonist whereas CBD is known as a moderately potent antagonist at both the CB_1_ and CB_2_ receptors [15,17,18]. Similarly, *Δ^9^*-THCA exhibits agonist activity at CB_1_ and CB_2_, although its affinities for these receptors are several orders of magnitude lower than those of *Δ^9^*-THC and CBD. In contrast, CBDA shows little to no binding at either cannabinoid receptor [15,19]. One of the interesting findings in research into cannabis and cannabis-derived extracts is that the combined biological and physiological actions of these phytocannabinoids when consumed as extracts are quite different to those of the pure compounds, even when considering cannabis extracts enriched in a particular phytocannabinoid. Here, using 59 cannabis samples consumed by patients, we were interested in understanding the collective response of the phytocannabinoids (and potentially other bioactive compounds) in these cannabis extracts. We initially limited our efforts to the four most abundant and studied phytocannabinoids, *Δ^9^*-THC, *Δ^9^*-THCA, CBD, and CBDA, to gain an understanding of potential relationships with routinely measurable receptor activities using PLS methods.

One of the main advantages of PLS regression is its ability to account for multi-collinearity among the independent variables, i.e., the concentrations of the phytocannabinoids in the samples. This is especially pertinent with regards to medical cannabis, for which the phytocannabinoids are produced through a multi-branched biosynthetic pathway [2]. The four phytocannabinoids in the cannabis samples considered in this study (*Δ^9^*-THCA, CBDA, *Δ^9^*-THC, and CBD) are synthesized through a combination of enzyme catalysis and exposure to environmental heat, and undergo various changes during processing and storage until they are consumed [20,21,22,23,24]. CBDA and *Δ^9^*-THCA, and by extension CBD and *Δ^9^*-THC, lie on separate branches of the phytosynthetic pathway originating from a common precursor, cannabigerolic acid (CBGA). A negative correlation, thus, can be observed between the total *Δ^9^*-THC content and total CBD content in the cannabis samples. Indeed, regression of our quantitative data revealed strong negative correlations between total *Δ^9^*-THC and total CBD contents in both dried cannabis and cannabis oil sample groups (Table 3A,B, respectively). Further breakdown of the cannabis samples reveals that a stronger correlation exists between the CBD and *Δ^9^*-THC concentrations when extracted with MAE, as well as a stronger correlation between CBDA and *Δ^9^*-THCA concentrations of SFE samples (Table 3C,D, respectively).

The first model (Equation (1)) suggests that increasing the concentrations of CBD and *Δ^9^*-THC increases the potency of the cannabis extracts as CB_2_ receptor agonists, whereas increasing the CBDA and *Δ^9^*-THCA concentrations decreases the corresponding potency. The coefficient of determination (*r*^2^) and *Q*^2^ statistics, 0.842 and 0.784, respectively, for this model indicate a strong correlation between the dependent and independent variables, as well as good predictive power for the equation. Interestingly, CBD, a known antagonist at CB_2_ receptor, exhibits CB_2_ agonism when administered as part of a cannabis extract where the other three phytocannabinoids are in smaller proportions. Furthermore, the relative values of the variable coefficients in Equation (1) show that CBD has a higher CB_2_ receptor potency among the four phytocannabinoids—two orders of magnitude greater than that of *Δ^9^*-THC, which exhibits the highest individual CB_2_ receptor activity. Such pronounced pharmacodynamic differences may be due to synergistic and possibly antagonistic interplay between the ligands at the cannabinoid receptors, where simultaneous occupation of the orthosteric and allosteric sites by multiple ligands in the mixture of compounds leads to unique pharmacological responses [15]. This phenomenon of inter-modulation between phytocannabinoids observed in medical cannabis and cannabis formulations has been collectively termed *the entourage effect* [13]. Here, in the context of CB_2_ receptor agonism, we stress that the combined effects of complex natural products, such as cannabis extracts, can be described by mathematical models with high correlation using the concentrations of individual quantifiable compounds, as shown in this study.

The model described by Equation (1) also lends weight to the hypothesis that medical cannabis and cannabinoid-based products show efficacy for neuropathic pain management [25,26]. In contrast to the CB_1_ receptor, which is mainly found in the central nervous system (CNS), the CB_2_ receptor is primarily expressed in peripheral tissues, especially those belonging to the immune system [27]. Activation of the CB_2_ receptor pathway has been shown to modulate nociception in animal models [28,29,30]. Although none of the four phytocannabinoids quantified in the current study are known to behave as CB_2_ agonists individually, phytocannabinoid synergy at the CB_2_ receptor may result in CB_2_ agonism and, in turn, the subsequent anti-nociceptive effects observed clinically. This may explain the anti-nociceptive effects observed in cannabis plant extracts [31,32,33], as well as CBD-containing cannabinoid mixtures, such as nabiximols, unlike CBD alone [34].

The second model, represented by Equation (2), shows that increasing CBD and *Δ^9^*-THC concentrations decreases their potency as CB_1_ receptor agonists, whereas increasing CBDA and *Δ^9^*-THCA concentrations increases the potency of cannabis oils. Similar to the trend observed in model 1, CBD concentration exerts the largest effect among the four variables on receptor affinity; additionally, among the three models the third is the only model that attributes receptor activity solely to the cannabinoid acids (CBDA and *Δ^9^*-THCA), which show weaker or no CB_1_ affinity. It is noteworthy that Equation (3) describes a similar relationship with cannabis extracts, rather than cannabis oils, and projects a different result whereby CB_1_ receptor activity is entirely attributed to the concentration of *Δ^9^*-THC. Although arguments can be made for the entourage effect, it is unlikely that a change in the form of dosing (from extracts to oils) alone could lead to such drastic differences in cannabinoid pharmacodynamics at the CB_1_ receptor [35,36]. Instead, this is likely the result of model inadequacy, brought about by the small sample size (9) that was used to construct Equation (2). Indeed, although the coefficient of determination (*r*^2^ = 0.679) indicates moderate correlation, the *Q*^2^ parameter are much smaller (*Q*^2^ = 0.304), and this difference is indicative of the poor predictive power of the model. As such, the incorporation of additional data from the remaining patients may serve to reveal the true nature of this relationship.

As mentioned above, the third model represented by Equation (3) shows that increasing *Δ^9^*-THC concentration increases potency, whereas increasing concentrations of CBD, CBDA, and *Δ^9^*-THCA lower the combined potency of cannabis extracts as agonists at the CB_1_ receptor. This relationship is consistent with the individual pharmacological behavior of the four phytocannabinoids at the CB_1_ receptors. This also corroborates observations that CBD modulates the acute psychoactive properties of *Δ^9^*-THC in vivo, as well as having potential for psychosis, after cannabis consumption [27,37,38]. However, the coefficient of determination (*r*^2^ = 0.204) suggests a poor correlation at best between these two variables. Furthermore, the leave-one-out (LOO) cross-validated *r*^2^ value (*Q*^2^), was also relatively low (*Q*^2^ = 0.140). Although the sample size is large (*n* = 42), this model yielded the poorest correlation between cannabinoid receptor agonist activity and the concentrations of the four phytocannabinoids in the corresponding cannabis samples. However, the closeness of the *r*^2^ and *Q*^2^ parameters suggests that the model may have high predictive quality.

Comparison between predicted and experimental receptor potencies for Equation (3) (Figure 2c) revealed four outliers corresponding to samples #2, #4, #12, and #13 (Table 1). Exclusion of these outliers generated the following model (Equation (4)) for the CB_1_ agonism of cannabis extracts:

Log(EC_50_) = −1.684 × 10^−4^ CBD − 6.018 × 10^−3^*Δ^9^*-THC + 1.240 × 10^−4^ CBDA^2^ + 6.966 × 10^−3^*Δ^9^*-THCA + 2.602, (4)

where *n* = 38, *r*^2^ = 0.422, *Q*^2^ = 0.367, RMSE = 0.387, Components = 1.

In addition to an improvement in correlation strength, the pharmacodynamics of CBD are reversed in the new model, where increasing CBD concentrations is shown to improve CB_1_ agonist potency, albeit to a lesser extent than *Δ^9^*-THC. Similar to the results revealed by Equation (1), the pharmacology of CBD when administered as part of a cannabis extract is once again pronouncedly different from that of CBD alone. As shown by its *Q*^2^ value, this model maintains good predictive quality. Among the outliers, samples #2 and #12 were from high-THC cannabis samples, whereas samples #4 and #13 contained significant amounts of CBD and *Δ^9^*-THC. The chemical profiles of the four quantified phytocannabinoids are not anomalous in these four outliers. However, it is possible that potentially high concentrations of yet-to-be-quantified phytochemicals may have influenced the affinities of these four outliers for the CB_1_ receptors.

To evaluate the utility of the above models, we simulated the EC_50_ values of the four quantified phytocannabinoids at the CB_1_ receptor (Equations (2)–(4)) and CB_2_ receptor (Equation (1)) as agonists. Thus, the 100% concentration of each test phytocannabinoid along with null concentrations of the remaining three phytocannabinoids yielded the predicted receptor potencies (Table 4). These predictions were then compared against experimental EC_50_ values determined in the same assays [13]. The predicted EC_50_ values from Equation (1) for *Δ^9^*-THCA and CBDA, Equation (3) for *Δ^9^*-THC, CBD, *Δ^9^*-THCA, and CBDA and Equation (4) for CBD, *Δ^9^*-THCA, and CBDA are within an order of magnitude from their experimental counterparts. In particular, the predicted EC_50_ values for *Δ^9^*-THCA at CB_1_ (Equations (3) and (4)) and for CBDA at CB_2_ (Equation (1)) differ by <10% from their experimental values. Thus, with the incorporation of additional training data, these models can be further optimized to estimate the in vitro activity of any cannabis extract with accuracy.

However, predictions from these models may be limited by the trends and the size of the training datasets. For example, Equations (1), (3) and (4) are applicable to extracts obtained from cannabis flowers, in which the concentration of the most abundant phytocannabinoid consistently exceeds 25% (*w*/*w*). On the other hand, Equation (2) may be used for cannabis derivative oil products, in which the phytocannabinoid content generally does not exceed 10% (*w*/*v*) for any quantified phytocannabinoid. Additionally, Schüürmann et al. noted that the derivation for *Q*^2^ in the LOO methodology (i.e., where *Q*^2^ is calculated based on the training set rather than the test set) tends to overestimate *Q*^2^ when observations lie mostly at the two extremes of the dependent variable range, a situation that may apply to Equation (1) [39,40].

Another caveat when interpreting the models lies in their constant terms, which are up to four magnitudes of order larger than the coefficients of the independent variables. This captures the effects of unquantified factors on receptor potency, including minor cannabinoids and non-cannabinoid phytochemicals in this case. The rationale for selecting the CBD and *Δ^9^*-THC compound families as independent variables lies in their relative abundance because total CBD and *Δ^9^*-THC concentrations in the cannabis plant are generally many times higher than those of other phytocannabinoids [41] and are, thus, thought to be the primary determinants of cannabinoid receptors activities. However, complex natural products such as cannabis can contain up to hundreds of unique compounds, and it remains unknown how less abundant phytocannabinoids, such as cannabichromene (CBC) and cannabigerol (CBG), affect the activity of cannabis extracts at cannabinoid receptors [42,43].

## 4. Materials and Methods

### 4.1. General Information

Milli-Q purified water and high-performance liquid chromatography (HPLC) grade methanol were used for chromatographic analyses. All other commercial solvents and reagents were used without further processing. Liquid CO_2_ (SFE grade) for supercritical fluid extraction was purchased from Praxair^®^ (Scarborough, ON, Canada). Analytical standards for *Δ^9^*-THCA, CBDA, *Δ^9^*-THC, CBD, and *Δ^9^*-THC-d_3_ were purchased from Sigma-Aldrich^®^ Canada (Oakville, ON, Canada) as Certified Reference Standards. Tissue culture media, fetal bovine serum, and trypsin were obtained from Gibco™ (ThermoFischer Scientific, Ottawa, ON, Canada). HitHunter cAMP assay kit was obtained from DiscoverX Corporation (Fremont, CA, USA). All other chemicals were obtained from Sigma-Aldrich and were of analytical grade and used as such.

### 4.2. Cannabis Samples

Samples were collected from patients consuming cannabis for medical reasons. These patients were recruited as a part of two clinical studies investigating the use of medical cannabis in chronic pain (University Health Network REB#: 16-6375) and PTSD (University Health Network REB#: 17-5180.0; Center for Addiction and Mental Health REB#: 036/2017). A study visit was arranged for eligible participants who had passed clinical study screening. During the visit, a sample of medical cannabis products consumed by each participant (200 mg of dried medical cannabis or 0.2 mL of cannabis oil) was collected for analysis. The cannabis samples were transported to a designated secure area by qualified personnel for further processing and analysis.

### 4.3. Extraction of Cannabis for Cannabinoids 

Cannabis samples in their oil form were analyzed for their chemical content without further processing. Cannabis samples collected as dried plant material were extracted using either supercritical fluid extraction (SFE) or microwave assisted extraction (MAE) following previously described procedures [2,15,44] and the extracts were subjected to chemical analyses. A total of 59 cannabis samples are included in this investigation: 45 dried flower samples, two resins, and 12 oil samples. Samples 1 and 2 were obtained as resins and analyzed as received, samples 3–35 were processed using MAE, and samples 36–47 were processed using SFE (Table 1).

### 4.4. Chemical Analysis

The cannabis extracts were analyzed by liquid chromatography-mass spectrometry (LC-MS) on an ACQUITY UPLC H-Class system (Waters^®^ Limited, Mississauga, ON, Canada) equipped with Quaternary Solvent Manager and Sample Manager FTN. A Waters^®^ MS 3100 mass spectrometer was used to monitor the samples. Chromatographic separation was achieved on an Acquity UPLC^®^ BEH column (2.1 × 50 mm, C18, 1.7 µm). The sample injection plate and the column were maintained at 15 and 40 °C, respectively. The injection volume was 10 µL. *Δ^9^*-THC-d_3_ was added to samples as an internal standard to monitor the sensitivity of the UPLC-MS system. Working stock solutions of the extracts and cannabis oil samples were prepared in ethanol and methanol, respectively.

Analytical samples were prepared by diluting defined amounts of stock solutions in H_2_O/MeOH (3:7) with 0.1% formic acid (mobile phase I), filtering (Millex-GV^®^ Syringe Filters, 0.22 µm; EMD Millipore, Oakville, ON, Canada), and further diluting with mobile phase I as needed. The internal standard was then added before analytical samples were injected into the UPLC system. The mobile phase for LC-MS consisted of H_2_O (A) and MeOH (B), with 0.1% formic acid. The gradient was programmed as follows: 0–4.5 min (30% A/70% B to 0% A/100% B, linear gradient), 4.5–5.0 min (0% A/100% B, isocratic), 5.0–5.2 min (0% A/100% B to 30% A/70% B, linear gradient), and 5.2–6 min (30% A/70% B, isocratic). The flow rate was 0.6 mL/min, and all samples were analyzed in triplicate. The mass scan in the range of 150–500 *m*/*z*, and single ion recordings (SIRs) in positive and negative modes (+ve = 287.20, 311.20, 315.23, 317.25, 318.24, 331.47, 345.45; −ve = 329.46, 343.45, 357.21, 359.22 *m*/*z*) were monitored.

When quantifying the phytocannabinoids present at relatively low abundances (i.e., <5% *w*/*w* and 1% *w*/*v* in extracts and oils, respectively), analytical samples were prepared by diluting defined amounts of stock solutions in H_2_O/MeOH (4:6) with 0.1% formic acid (mobile phase II), filtering (Millex-GV^®^ Syringe Filters, 0.22 µm; EMD Millipore), and further diluting with mobile phase II as needed. The internal standard was then added before analytical samples were injected into the UPLC system. The mobile phase for LC-MS consisted of H_2_O (A) and MeOH (B), with 0.1% formic acid. The gradient was programmed as follows: 0–13.5 min (40% A/60% B to 0% A/100% B, linear gradient), 13.5–14.0 min (0% A/100% B, isocratic), 14.0–14.2 min (0% A/100% B to 40% A/60% B, linear gradient), and 14.2–15 min (30% A/70% B, isocratic). The flow rate was 0.5 mL/min, and all samples were analyzed in triplicate. Single ion recordings (SIRs) in positive and negative modes (+ve = 287.20, 311.20, 315.23, 317.25, 318.24; −ve = 329.46, 357.21, 359.22 *m*/*z*) were monitored.

All SIR chromatograms were analyzed using Waters^®^ Empower3^®^, and the concentration of each phytocannabinoid was determined using its corresponding standard curve plotted in Grafit^®^ 5.0.10 (Erithacus Software Limited, East Grinstead, West Sussex, United Kingdom) using the following expression:

% (*w*/*w*) or % (*w*/*v*) Phytocannabinoid = [(Concentration) × (Dilution Factor) × 100]/(Sample Stock Solution Concentration).

All injections were performed in triplicate, and all reported values are averaged values. The lower limits of detection (LLD) and lower limits of quantitation (LLQ) for the quantified phytocannabinoids are reported in Table S1.

### 4.5. CB_1_ and CB_2_ Receptor Assays 

The cannabis samples extracts and cannabis oils were assayed against CB_1_ and CB_2_ receptors on a cAMP reporter platform using CHO-K_1_-CB_1_ and CHO-K_1_-CB_2_ cell lines, respectively (DiscoverX Corporation), according to previously described methods [15]. The efficacies of the cannabis extracts were calculated as the percentage inhibition of forskolin-stimulated cAMP production. CP-55940 was used as a reference agonist at CB_1_ and CB_2_ receptors, whereas SR-141716 and AM630 were used as reference antagonists at CB_1_ and CB_2_ receptors, respectively. The data were analyzed according to previously described methods [15]. Graphpad^®^ Prism 7 (GraphPad Software, San Diego, CA, USA) was used to derive dose-response curves for each sample. Concentration of the major phytocannabinoid in each cannabis extract (as shown in Table 1 and Table 2) was used to fit and derive EC_50_ and IC_50_ for each cannabis sample. For example, for cannabis sample 1, *Δ*^9^-THC concentration was used to derive the dose-response profile and the corresponding EC_50_ or IC_50_ value.

### 4.6. Statistical Analysis

A multivariate PLS analysis was employed to evaluate potential relationships between receptor responses (agonism and antagonism at the CB_1_ and CB_2_ receptors) and chemical composition (*Δ^9^*-THCA, CBDA, *Δ^9^*-THC, and CBD). Normalized potencies of the extracts at each receptor (log(EC_50_) or log(IC_50_), nM) were correlated against the concentrations of four major phytocannabinoids (% *w*/*w* or % *w*/*v*) on the XLSTAT^®^ 2019 platform (Addinsoft, Paris, France) for Microsoft Excel^®^ (Microsoft Corporation, Redmond, WA, USA) Because of differences in measurement units, the dried plant samples (concentrations in % *w*/*w*) and oil samples (concentrations in % *w*/*v*) were correlated separately. Before analysis, the data were categorized into eight subsets based on sample type (dried plant/cannabis oil), receptor subtype (CB_1_/CB_2_) and assay mode (agonism/antagonism) (Table S2). Extracts that were inactive in the receptor assays or whose EC_50_/IC_50_ values could not be precisely derived were excluded from the analysis. Groups with *n* < 5 samples were deemed to not be statistically significant and excluded from the analysis. For each remaining group, prediction models were generated using a combination of the linear explanatory variables *Δ^9^*-THCA, CBDA, *Δ^9^*-THC, and CBD, as well as the quadratic variables *Δ^9^*-THCA^2^, CBDA^2^, *Δ^9^*-THC^2^, and CBD^2^, where models were optimized to maximize *r*^2^ while minimizing root mean square error (RMSE) and the value of the constant term (Tables S3–S5). All models were also cross-validated using the LOO technique, allowing for the evaluation of predictive quality through the *Q*^2^ statistic, which is the LOO-cross-validated *r*^2^ value [40]. In general, a difference of less than 0.2 between the *r*^2^ and *Q*^2^ parameters is indicative of good predictive power for a mathematical model [45]. PLS regression captures factors that describe most of the relationship between the dependent and independent variables while preventing the incorporation of superfluous factors that lower the predictive power of models [46,47].

## 5. Conclusions

A first-of-its-kind QSAR-like quantitative analysis was performed on 59 samples of cannabis extracts and cannabis oils to elucidate the relationships between the concentrations of phytocannabinoids in the cannabis extracts and the corresponding biological activities at the CB_1_ and CB_2_ receptors. To achieve this, we employed PLS regression analyses. These models can be useful for predicting the potency of (1) CB_1_ and (2) CB_2_ agonism in cannabis extracts; however, (3) CB_1_ agonism in cannabis oils will likely require a larger training dataset to generate accurate predictions. Our analysis of the statistical parameters suggest insignificant relationships between the major phytocannabinoid content and receptor affinity for CB_1_ but good correlative models at CB_2_ receptors with the current dataset. The models disclosed here for the CB_1_ and CB_2_ agonism of cannabis extracts reliably predicted the potencies of single phytocannabinoids, which were comparable to the experimentally derived potencies in turn validating the models. It must also be noted that some phytocannabinoids such as CBD are considered “safe” by many people although any chemical compound including CBD [48] can exhibit adverse effects leading to toxicities; data and results presented in this study do not consider any such effects. Note that one can include other compounds present at physiologically significant concentrations from the cannabis extracts in the description of the chemical composition in each equation, and future work will include such expansion. This is the first study disclosing a relationship between the major phytocannabinoids concentrations in cannabis extracts and the corresponding cannabinoid receptors activities. The strategies described in this work and further refinement utilizing larger sample sets and additional variables may further improve the accuracy to understand the effects of cannabis extracts on cannabinoid receptors.

## Figures and Tables

**Table 1:** Phytocannabinoid concentrations and corresponding receptor potencies of cannabis extracts, as well as pure phytocannabinoids and reference compounds.

Sample	Phytocannabinoid Concentration (% *w*/*w*)				Receptor Potency (μM)				Normalized Receptor Potency (nM)			
					^a^ EC_50_ (Agonist)		^b^ IC_50_ (Antagonist)		Log(EC_50_)		Log(IC_50_)	
	CBD	*Δ^9^*-THC	CBDA	*Δ^9^*-THCA	CB_1_	CB_2_	CB_1_	CB_2_	CB_1_	CB_2_	CB_1_	CB_2_
CP-55940 ^3^	-	-	-	-	0.0025 ± 0.0009	0.0026 ± 0.0004	-	-	0.398	0.415	-	-
SR-141716 ^3^	-	-	-	-	-	-	0.024 ± 0.003	-	-	-	1.380	-
AM-630 ^3^	-	-	-	-	-	-	-	0.146 ± 0.084	-	-	-	2.164
CBD ^3^	100	-	-	-	10 ± 2	>10	>10	7 ± 2	4.000	-	-	3.845
*Δ^9^*-THC ^3^	-	100	-	-	0.015 ± 0.001	>10	>10	1.4 ± 0.3	1.176	-	-	3.146
CBDA	-	-	100	-	17 ± 3	17 ± 4	>10	>10	4.230	4.230	-	-
*Δ^9^*-THCA ^3^	-	-	-	100	1.8 ± 0.7	30 ± 15	>10	>10	3.255	4.477	-	-
1	0.4 ± 0.04	49 ± 0.9	0	0	0.05 ± 0.02	>100	>1	>10	1.675	-	-	-
2	1.05 ± 0.02	29 ± 0.2	0	0	0.013 ± 0.008	>7	>10	>1	1.114	-	-	-
3	0.169 ± 0.007	39.2 ± 0.8	0	0.036 ± 0.005	1.3 ± 0.5	>100	>1	>100	3.117	-	-	-
4	27.9 ± 0.3	11.5 ± 0.2	0	0	30 ± 10	>100	0.13 ± 0.06	>10	4.479	-	2.117	-
5	33.0 ± 0.8	0	0	0	0.2 ± 0.1	>10	>1	>1	2.332	-	-	-
6	0.163 ± 0.006	39 ± 1	0	0	0.1 ± 0.1	>33	>1	>10	2.152	-	-	-
7	0.135 ± 0.003	35.9 ± 0.3	0	0	>10	>10	>1	>10	-	-	-	-
8	10.5 ± 0.3	23.4 ± 0.9	0	0	>10	>10	>1	2.5 ± 0.8	-	-	-	3.401
9	0.163 ± 0.003	70.0 ± 0.9	0	0	0.2 ± 0.1	>2	>2	>19	2.332	-	-	-
10	0.153 ± 0.002	73.1 ± 0.9	0	0	0.4 ± 0.3	>13	6 ± 2	>19	2.651	-	3.779	-
11	0.159 ± 0.010	72.5 ± 0.3	0	0	0.7 ± 0.3	>13	>2	>0.2	2.846	-	-	-
12	0	41.8 ± 0.3	0	0	6 ± 2	>10	>1	>10	3.803	-	-	-
13	21.9 ± 0.7	36.2 ± 0.5	0	0	9 ± 6	>48	>1	>10	3.978	-	-	-
14	0	42.2 ± 0.6	0	0	0.5 ± 0.4	>100	>1	>10	2.696	-	-	-
15	51.7 ± 0.4	0	0	0	0.8 ± 0.4	>100	>10	>1	2.926	-	-	-
16	17.6 ± 0.5	41.1 ± 0.7	0	0	0.2 ± 0.1	>99	>1	>10	2.318	-	-	-
17	0	28.2 ± 0.4	0	0	0.2 ± 0.1	>100	>1	1.3 ± 0.8	2.365	-	-	3.102
18	0	17.4 ± 0.9	0	0	0.2 ± 0.1	>100	>1	>10	2.362	-	-	-
19	23.9 ± 0.3	11.57 ± 0.10	0	0	0.11 ± 0.06	>10	>1	>1	2.060	-	-	-
20	18.1 ± 0.4	10.7 ± 0.6	0	0	>0.1	>10	>1	>1	-	-	-	-
21	0	33 ± 1	0	0	>0.1	>10	>1	>10	-	-	-	-
22	0	65.6 ± 0.5	0	0	0.19 ± 0.04	>100	>1	>10	2.279	-	-	-
23	0	39.4 ± 0.1	0	0	0.04 ± 0.04	>100	>1	1.6 ± 0.9	1.646	-	-	3.202
24	0	47.4 ± 0.5	0	0	0.15 ± 0.05	>100	>10	>10	2.187	-	-	-
25	0	54.9 ± 0.3	0	0	0.20 ± 0.09	>100	>10	>10	2.292	-	-	-
26	48.9 ± 0.6	0	0	0	0.5 ± 0.3	>100	>10	>1	2.676	-	-	-
27	0	48.6 ± 0.7	9.7 ± 0.3	7.6 ± 0.4	0.15 ± 0.08	3 ± 2	>1	>10	2.108	3.477	-	-
28	0.30 ± 0.01	55 ± 2	0	0	0.20 ± 0.08	0.2 ± 0.1	>10	>10	2.307	2.304	-	-
29	0.37 ± 0.02	44 ± 2	0	0	0.20 ± 0.09	>114	>11	>11	2.291	-	-	-
30	0	43.2 ± 0.1	0	0	0.19 ± 0.08	0.16 ± 0.06	>11	>10	2.290	2.215	-	-
31	0.37 ± 0.01	56 ± 1	0	0	0.3 ± 0.3	>100	>10	>1	2.442	-	-	-
32	0.378 ± 0.008	46 ± 1	0	0	>0.012	>12	>12	>12	-	-	-	-
33	0.282 ± 0.009	46 ± 1	0	0	0.12 ± 0.05	0.10 ± 0.07	>10	>10	2.079	2.015	-	-
34	0.44 ± 0.02	38.7 ± 0.8	0	0	0.3 ± 0.2	>100	>100	>100	2.402	-	-	-
35	0.77 ± 0.03	43 ± 1	0	0	0.15 ± 0.08	>10	>10	>0.1	2.166	-	-	-
36	0	0	49 ± 1	32.0 ± 0.3	3 ± 1	18 ± 7	>10	>10	3.544	4.262	-	-
37	0	0	0.092 ± 0.007	51.2 ± 0.6	3 ± 1	21 ± 11	>93	>0.09	3.490	4.323	-	-
38	0	0	28.3 ± 0.4	27.6 ± 0.2	0.08 ± 0.03	13 ± 5	>1	>11	1.878	4.098	-	-
39	0	0	0.050 ± 0.003	47.8 ± 0.8	1.3 ± 0.2	10 ± 2	>88	>7	3.109	3.999	-	-
40	0	0	60 ± 1	0	0.8 ± 0.3	15 ± 6	>9	>89	2.920	4.183	-	-
41	0	0	0.232 ± 0.005	61.5 ± 0.4	5 ± 3	9 ± 2	>103	>10	3.708	3.935	-	-
42	0	0	0.094 ± 0.010	62.2 ± 0.5	0.6 ± 0.2	6 ± 2	>94	>9	2.767	3.796	-	-
43	0	0	0.076 ± 0.002	64 ± 1	0.5 ± 0.2	46 ± 19	>10	>10	2.724	4.663	-	-
44	0	0	0	76.1 ± 0.3	0.5 ± 0.1	73 ± 59	>10	>10	2.738	4.860	-	-
45	0	0	52 ± 1	15.7 ± 0.02	2 ± 1	13 ± 5	>10	>10	3.358	4.100	-	-
46	0	0	0	54 ± 1	0.7 ± 0.4	12 ± 4	>10	>10	2.825	4.096	-	-
47	0	0	0	73 ± 3	4 ± 1	>10	>10	>10	3.564	-	-	-

^a^ Half-maximal effective concentrations. ^b^ Half-maximal inhibitory concentrations. ^3^ [15].

**Table 2:** Phytocannabinoid concentrations and corresponding receptor potencies of cannabis oil samples.

Sample	Phytocannabinoid Concentration (% *w*/*v*)				Receptor Potency (μM)				Normalized Receptor Potency (nM)			
					^a^ EC_50_ (Agonist)		^b^ IC_50_ (Antagonist)		Log(EC_50_)		Log(IC_50_)	
	CBD	*Δ^9^*-THC	CBDA	*Δ^9^*-THCA	CB_1_	CB_2_	CB_1_	CB_2_	CB_1_	CB_2_	CB_1_	CB_2_
1	2.05 ± 0.03	0.158 ± 0.007	0	0	>2	>2	>2	>0.02	-	-	-	-
2	0.0072 ± 0.0002	1.815 ± 0.010	0	0	0.03 ± 0.03	>2	>0.2	>0.2	1.513	-	-	-
3	1.168 ± 0.009	0.290 ± 0.007	0	0	0.04 ± 0.02	>1	>37	0.024 ± 0.008	1.642	-	-	1.385
4	0.427 ± 0.004	0.512 ± 0.006	0	0	0.03 ± 0.02	>0.8	>0.8	>0.08	1.516	-	-	-
5	1.71 ± 0.02	0.112 ± 0.001	0	0	0.2 ± 0.2	>2	>2	>2	2.287	-	-	-
6	3.79 ± 0.09	0.199 ± 0.006	0	0	0.20 ± 0.08	>13	>13	>13	2.296	-	-	-
7	8.9 ± 0.2	0	0	0	0.5 ± 0.3	>10	>10	>10	2.711	-	-	-
8	7.5 ± 0.1	4.6 ± 0.1	31.7 ± 0.6	15.3 ± 0.3	0.3 ± 0.2	>0.001	>10	>10	2.304	-	-	-
9	0.0133 ± 0.0003	4.1 ± 0.2	0	0	0.3 ± 0.2	>0.009	>0.9	>9	2.401	-	-	-
10	5.4 ± 0.1	0	0	0	>0.1	>0.001	>1	>1	-	-	-	-
11	2.73 ± 0.02	0.173 ± 0.003	1.34 ± 0.03	0.017 ± 0.003	0.04 ± 0.02	>3	>30	>3	1.391	-	-	-
12	1.258 ± 0.008	0.909 ± 0.006	0.035 ± 0.001	0.0017 ± 0.0001	>0.001	0.0015 ± 0.0007	>4	>4	-	0.162	-	-

^a^ Half-maximal effective concentrations. ^b^ Half-maximal inhibitory concentrations.

**Table 3:** Intra-variable correlation (Pearson’s correlation coefficients) between major cannabinoid concentrations in the cannabis extracts from dried cannabis (**A**), cannabis oils (**B**), cannabis extracts using MAE (**C**) and SFE (**D**). Note: Total *Δ^9^*-THC = *Δ^9^*-THC + *Δ^9^*-THCA Content; Total CBD = CBD + CBDA Content.

	CBD	*Δ^9^*-THC	CBDA	*Δ^9^*-THCA	Total *Δ^9^*-THC	Total CBD
**A**						
**CBD**	1					
***Δ^9^*** **-THC**	−0.330	1				
**CBDA**	−0.137	−0.344	1			
***Δ^9^*** **-THCA**	−0.230	−0.613	0.056	1		
**Total *Δ^9^*-THC**	0.615	−0.513	0.697	−0.122	1	
**Total CBD**	−0.637	0.443	−0.328	0.437	−0.722	1
**B**						
**CBD**	1					
***Δ^9^*** **-THC**	0.002	1				
**CBDA**	0.489	0.675	1			
***Δ^9^*** **-THCA**	0.489	0.680	0.999	1		
**Total *Δ^9^*-THC**	0.681	0.568	0.972	0.971	1	
**Total CBD**	0.383	0.819	0.976	0.978	0.923	1
**C**						
**CBD**	1					
***Δ^9^*** **-THC**	−0.750	1				
**CBDA**	−0.091	0.094	1			
***Δ^9^*** **-THCA**	−0.091	0.094	1.000	1		
**Total *Δ^9^*-THC**	0.993	−0.742	0.025	0.024	1	
**Total CBD**	−0.750	0.998	0.159	0.159	−0.734	1
**D**						
**CBD**	1					
***Δ^9^*** **-THC**	N/A	1				
**CBDA**	N/A	N/A	1			
***Δ^9^*** **-THCA**	N/A	N/A	−0.910	1		
**Total *Δ^9^*-THC**	N/A	N/A	1	−0.910	1	
**Total CBD**	N/A	N/A	−0.910	1	−0.910	1

**Table 4:** Predicted and experimental EC_50_ values for four major phytocannabinoids at CB_1_ and CB_2_ receptors. Generally, a difference of less than 0.2 between *r*^2^ and *Q*^2^ is indicative of good predictive power for models. Note: Pred—Predicted, Exp—Experimental.

Cannabinoid	EC_50_, CB_2_ Agonism (μM)		EC_50_, CB_1_ Agonism (μM)					
	Equation (1) (*r*^2^ = 0.842, *Q*^2^ = 0.784)		Equation (2) (*r*^2^ = 0.679, *Q*^2^ = 0.304)		Equation (3) (*r*^2^ = 0.204, *Q*^2^ = 0.140)		Equation (4) (*r*^2^ = 0.422, *Q*^2^ = 0.367)	
	Pred	Exp	Pred	Exp	Pred	Exp	Pred	Exp
CBD	<10^−290^	>10	>10^12^	10 ± 2	1.7	10 ± 2	0.4	10 ± 2
*Δ^9^*-THC	0.13	>10	>10^500^	0.015 ± 0.001	0.12	0.015 ± 0.001	0.100	0.015 ± 0.001
CBDA	18.79	17 ± 4	1.4 × 10^−4^	17 ± 3	5.9	17 ± 3	7	17 ± 3
*Δ^9^*-THCA	131.83	30 ± 15	4.8 × 10^−6^	1.8 ± 0.7	1.9	1.8 ± 0.7	2	1.8 ± 0.7

## Supplementary Materials

The following are available online, Table S1: LLD and LLQ values for the phytocannabinoids quantified in this study; Table S2: Categorization of data prior to regression analysis; Table S3: Preliminary prediction models generated using all combinations of linear and quadratic explanatory variables, describing the relationship between chemical composition and potency at CB_2_ as agonists for extracts obtained from dried plant samples; Table S4: Preliminary prediction models generated using all combinations of linear and quadratic explanatory variables, describing the relationship between chemical composition and potency at CB_1_ as agonists for cannabis-derivative samples; Table S5: Preliminary prediction models generated using all combinations of linear and quadratic explanatory variables, describing the relationship between chemical composition and potency at CB_1_ as agonists for extracts obtained from dried plant samples; Figure S1: Dose-response curves for CBD, CBDA, *Δ^9^*-THC and *Δ^9^*-THCA as agonists and antagonists at CB_1_ receptor; Figure S2: Dose-response curves for CBD, CBDA, *Δ^9^*-THC and *Δ^9^*-THCA as agonists and antagonists at CB_2_ receptor.
