# Supplementary material for: Bioactive Chemical Composition of Cannabis Extracts and Cannabinoid Receptors
Source: Molecules. 2020 Jul 30;25(15):3466. doi: 10.3390/molecules25153466 (PMC7436063; doi:10.3390/molecules25153466)

Manuscript ID: molecules-875053

Supplementary Material

Bioactive chemical composition of cannabis extracts and cannabinoid receptors

Yi Yang[1,2], Rupali Vyawahare[2], Melissa Lewis-Bakker[2], Hance A. Clarke[3], Albert H.C. Wong[4], and Lakshmi P. Kotra[1,2,5*]

[1]Department of Pharmaceutical Sciences, Leslie Dan Faculty of Pharmacy, University of Toronto, Ontario, M5S 3M2, Canada

[2]Centre for Molecular Design and Preformulations, and Krembil Research Institute, University Health Network, Toronto, Ontario, M5G 1L7, Canada

3Department of Anesthesia and Pain Management, Toronto General Hospital, University Health Network, Toronto, Ontario, M5G 1L7, Canada

[4]Campbell Family Mental Health Research Institute, Centre for Addiction and Mental Health, and the Departments of Psychiatry and Pharmacology, Faculty of Medicine, University of Toronto, Toronto, Ontario, M5T 1R8, Canada

[5]Multi-Organ Transplant Program, Toronto General Hospital, Toronto, Ontario, M5G 1L7, Canada

Table S1. LLD and LLQ values for the phytocannabinoids quantified in this study.

Phytocannabinoid	LLD (ng/ml)	LLQ (ng/ml)
Δ^9-THC	0.53 – 29.43	1.90 – 32.66
Δ^9-THCA	0.28 – 6.30	0.83 – 8.03
CBD	0.10 – 9.96	2.43 – 15.38
CBDA	0.62 – 7.59	1.17 – 9.04

Table S2. Categorization of data prior to regression analysis.

**Independent Variables: Cannabinoid Concentration (% w/w or % w/v or % w/w^2 or % w/v^2)**				**Dependent Variable: Receptor Potency (nM)**		
				**Sample Type**	**Receptor Subtype**	**Assay Mode**
CBD Or CBD^2	THC Or THC^2	CBDA Or $CBDA^2$	THCA Or $THCA^2$	Cannabis Extracts	CB_1	Agonism
						Antagonism
					CB_2	Agonism
						Antagonism
				Cannabis Oil	CB_1	Agonism
						Antagonism
					CB_2	Agonism
						Antagonism

Table S3: Preliminary prediction models generated using all combinations of linear and quadratic explanatory variables, describing the relationship between chemical composition and potency at CB_2 as agonists for extracts obtained from dried plant samples. The model chosen as Equation 1 is underlined.

Model #	Equation	r^2	RMSE	Q^2
1	$Log(EC_{50}) = -2.782\ CBD - 1.546 * 10^{-2}\ THC + 4.823 * 10^{-3}\ CBDA + 1.004 * 10^{-2}\ THCA + 3.664$	0.841	0.339	0.786
2	$Log(EC_{50}) = -2.779\ CBD - 1.545 * 10^{-2}\ THC + 8.449 * 10^{-5}\ CBDA^2 + 1.003 * 10^{-2}\ THCA + 3.673$	0.837	0.343	0.783
3	$Log(EC_{50}) = -2.967\ CBD - 1.649 * 10^{-2}\ THC + 5.143 * 10^{-3}\ CBDA + 1.363 * 10^{-4}\ THCA^2 + 3.765$	0.842	0.338	0.784
4	$Log(EC_{50}) = -2.957\ CBD - 1.644 * 10^{-2}\ THC + 8.990 * 10^{-5}\ CBDA^2 + 1.359 * 10^{-4}\ THCA^2 + 3.775$	0.836	0.344	0.779
5	$Log(EC_{50}) = -2.765\ CBD - 3.039 * 10^{-4}\ THC^2 + 4.794 * 10^{-3}\ CBDA + 9.982 * 10^{-3}\ THCA + 3.656$	0.820	0.360	0.754
6	$Log(EC_{50}) = -2.763\ CBD - 3.036 * 10^{-4}\ THC^2 + 8.398 * 10^{-5}\ CBDA^2 + 9.973 * 10^{-3}\ THCA + 3.666$	0.817	0.364	0.752
7	$Log(EC_{50}) = -2.943\ CBD - 3.234 * 10^{-4}\ THC^2 + 5.102 * 10^{-3}\ CBDA + 1.352 * 10^{-4}\ THCA^2 + 3.756$	0.819	0.361	0.749
8	$Log(EC_{50}) = -2.933\ CBD - 3.224 * 10^{-4}\ THC^2 + 8.917 * 10^{-5}\ CBDA^2 + 1.348 * 10^{-4}\ THCA^2 + 3.767$	0.814	0.367	0.746
9	$Log(EC_{50}) = -9.420\ CBD^2 - 1.546 * 10^{-2}\ THC + 4.823 * 10^{-3}\ CBDA + 1.004 * 10^{-2}\ THCA + 3.663$	0.838	0.342	0.783
10	$Log(EC_{50}) = -9.411\ CBD^2 - 1.545 * 10^{-2}\ THC + 8.450 * 10^{-5}\ CBDA^2 + 1.003 * 10^{-2}\ THCA + 3.673$	0.834	0.346	0.779
11	$Log(EC_{50}) = -10.045\ CBD^2 - 1.649 * 10^{-2}\ THC + 5.143 * 10^{-3}\ CBDA + * 1.363 * 10^{-4}\ THCA^2 + 3.764$	0.839	0.341	0.780
12	$Log(EC_{50}) = -10.012\ CBD^2 - 1.644 * 10^{-2}\ THC + 8.990 * 10^{-5}\ CBDA^2 + 1.359 * 10^{-4}\ THCA^2 + 3.774$	0.833	0.347	0.776
13	$Log(EC_{50}) = -9.355\ CBD^2 - 3.037 * 10^{-4}\ THC^2 + 4.790 * 10^{-3}\ CBDA + 9.974 * 10^{-3}\ THCA + 3.656$	0.817	0.364	0.749
14	$Log(EC_{50}) = -9.345\ CBD^2 - 3.034 * 10^{-4}\ THC^2 + 8.391 * 10^{-5}\ CBDA^2 + 9.964 * 10^{-3}\ THCA + 3.665$	0.813	0.367	0.747
15	$Log(EC_{50}) = -9.954\ CBD^2 - 3.231 * 10^{-4}\ THC^2 + 5.096 * 10^{-3}\ CBDA + 1.351 * 10^{-4}\ THCA^2 + 3.756$	0.816	0.365	0.743
16	$Log(EC_{50}) = -9.921\ CBD^2 - 3.220 * 10^{-4}\ THC^2 + 8.908 * 10^{-5}\ CBDA^2 + 1.346 * 10^{-4}\ THCA^2 + 3.766$	0.810	0.371	0.741

Table S4: Preliminary prediction models generated using all combinations of linear and quadratic explanatory variables, describing the relationship between chemical composition and potency at CB_1 as agonists for cannabis-derivative samples. The model chosen as Equation 2 is underlined.

Model #	Equation	r^2	RMSE	Q^2
1	$\boldsymbol{Log}(\boldsymbol{EC_{50}}) = 4.561 * 10^{-2}\ \boldsymbol{CBD} + 3.272 * 10^{-2}\ \boldsymbol{THC} + 4.849 * 10^{-3}\ \boldsymbol{CBDA} + 1.089 * 10^{-2}\ \boldsymbol{THCA} + 1.795$	0.264	0.394	-0.002
2	$\boldsymbol{Log}(\boldsymbol{EC_{50}}) = 4.484 * 10^{-2}\ \boldsymbol{CBD} + 3.217 * 10^{-2}\ \boldsymbol{THC} + 1.628 * 10^{-4}\ \boldsymbol{CBDA^2} + 1.071 * 10^{-2}\ \boldsymbol{THCA} + 1.798$	0.263	0.394	-0.015
3	$\boldsymbol{Log}(\boldsymbol{EC_{50}}) = 4.559 * 10^{-2}\ \boldsymbol{CBD} + 3.271 * 10^{-2}\ \boldsymbol{THC} + 4.847 * 10^{-3}\ \boldsymbol{CBDA} + 7.122 * 10^{-4}\ \boldsymbol{THCA^2} + 1.795$	0.264	0.394	-0.003
4	$\boldsymbol{Log}(\boldsymbol{EC_{50}}) = 4.482 * 10^{-2}\ \boldsymbol{CBD} + 3.215 * 10^{-2}\ \boldsymbol{THC} + 1.627 * 10^{-4}\ \boldsymbol{CBDA^2} + 7.001 * 10^{-4}\ \boldsymbol{THCA^2} + 1.798$	0.263	0.394	-0.015
<u>5</u>	$\boldsymbol{Log}(\boldsymbol{EC_{50}}) = 1.421 * 10^{-1}\ \boldsymbol{CBD} + 5.089 * 10^{-2}\ \boldsymbol{THC^2} - 2.356 * 10^{-2}\ \boldsymbol{CBDA} - 3.833 * 10^{-2}\ \boldsymbol{THCA} + 1.510$	0.679	0.260	0.304
6	$\boldsymbol{Log}(\boldsymbol{EC_{50}}) = 1.404 * 10^{-1}\ \boldsymbol{CBD} + 5.008 * 10^{-2}\ \boldsymbol{THC^2} - 6.453 * 10^{-4}\ \boldsymbol{CBDA^2} - 4.222 * 10^{-2}\ \boldsymbol{THCA} + 1.511$	0.664	0.266	0.211
7	$\boldsymbol{Log}(\boldsymbol{EC_{50}}) = 1.420 * 10^{-1}\ \boldsymbol{CBD} + 5.088 * 10^{-2}\ \boldsymbol{THC^2} - 2.362 * 10^{-2}\ \boldsymbol{CBDA} - 2.492 * 10^{-3}\ \boldsymbol{THCA^2} + 1.510$	0.679	0.260	0.303
8	$\boldsymbol{Log}(\boldsymbol{EC_{50}}) = 4.214 * 10^{-2}\ \boldsymbol{CBD} + 9.443 * 10^{-3}\ \boldsymbol{THC^2} + 1.529 * 10^{-4}\boldsymbol{CBDA^2} + 6.582 * 10^{-4}\ \boldsymbol{THCA^2} + 1.806$	0.275	0.391	-0.082
9	$\boldsymbol{Log}(\boldsymbol{EC_{50}}) = 5.424 * 10^{-3}\ \boldsymbol{CBD^2} + 3.327 * 10^{-2}\ \boldsymbol{THC} + 4.931 * 10^{-3}\ \boldsymbol{CBDA} + 1.107 * 10^{-2}\ \boldsymbol{THCA} + 1.829$	0.285	0.389	0.045
10	$\boldsymbol{Log}(\boldsymbol{EC_{50}}) = 5.330 * 10^{-3}\ \boldsymbol{CBD^2} + 3.270 * 10^{-2}\ \boldsymbol{THC} + 1.654 * 10^{-4}\ \boldsymbol{CBDA^2} + 1.088 * 10^{-2}\ \boldsymbol{THCA} + 1.831$	0.284	0.389	0.036
11	$\boldsymbol{Log}(\boldsymbol{EC_{50}}) = 5.421 * 10^{-3}\ \boldsymbol{CBD^2} + 3.326 * 10^{-2}\ \boldsymbol{THC} + 4.929 * 10^{-3}\ \boldsymbol{CBDA} + 7.242 * 10^{-4}\ \boldsymbol{THCA^2} + 1.829$	0.285	0.389	0.044
12	$\boldsymbol{Log}(\boldsymbol{EC_{50}}) = 5.328 * 10^{-3}\ \boldsymbol{CBD^2} + 3.268 * 10^{-2}\ \boldsymbol{THC} + 1.654 * 10^{-4}\ \boldsymbol{CBDA^2} + 7.117 * 10^{-4}\ \boldsymbol{THCA^2} + 1.831$	0.284	0.389	0.036
13	$\boldsymbol{Log}(\boldsymbol{EC_{50}}) = 1.339 * 10^{-2}\ \boldsymbol{CBD^2} + 1.440 * 10^{-2}\ \boldsymbol{THC^2} - 8.086 * 10^{-3}\ \boldsymbol{CBDA} - 1.306 * 10^{-2}\ \boldsymbol{THCA} + 1.752$	0.525	0.317	0.204
14	$\boldsymbol{Log}(\boldsymbol{EC_{50}}) = 1.323 * 10^{-2}\ \boldsymbol{CBD^2} + 1.385 * 10^{-2}\ \boldsymbol{THC^2} - 2.128 * 10^{-4}\ \boldsymbol{CBDA^2} - 1.392 * 10^{-2}\ \boldsymbol{THCA} + 1.753$	0.517	0.319	0.172
15	$\boldsymbol{Log}(\boldsymbol{EC_{50}}) = 1.339 * 10^{-2}\ \boldsymbol{CBD^2} + 1.439 * 10^{-2}\ \boldsymbol{THC^2} - 8.098 * 10^{-3}\ \boldsymbol{CBDA} - 8.481 * 10^{-4}\ \boldsymbol{THCA^2} + 1.752$	0.525	0.317	0.203

16	$\boldsymbol{Log}(\boldsymbol{EC_{50}}) = 1.323 * 10^{-2}\ \boldsymbol{CBD^2} + 1.384 * 10^{-2}\ \boldsymbol{THC^2} - 2.131 * 10^{-4}\ \boldsymbol{CBDA^2} - 9.039 * 10^{-4}\ \boldsymbol{THCA^2} + 1.753$	0.517	0.319	0.171

Table S5: Preliminary prediction models generated using all combinations of linear and quadratic explanatory variables, describing the relationship between chemical composition and potency at CB_1 as agonists for extracts obtained from dried plant samples. The model chosen as Equation 3 is underlined.

Model #	Equation	r^2	RMSE	Q^2
1	$\mathbf{Log}(\mathbf{EC_{50}}) = 5.435 * 10^{-3}\ \mathbf{CBD} - 6.101 * 10^{-3}\ \mathbf{THC} + 4.938 * 10^{-3}\ \mathbf{CBDA} + 5.834 * 10^{-3}\ \mathbf{THCA} + 2.682$	0.196	0.601	0.125
2	$\mathbf{Log}(\mathbf{EC_{50}}) = 5.479 * 10^{-3}\ \mathbf{CBD} - 6.151 * 10^{-3}\ \mathbf{THC} + 1.085 * 10^{-3}\ \mathbf{CBDA^2} + 5.882 * 10^{-3}\ \mathbf{THCA} + 2.681$	0.204	0.598	0.140
3	$\mathbf{Log}(\mathbf{EC_{50}}) = 5.690 * 10^{-3}\ \mathbf{CBD} - 6.388 * 10^{-3}\ \mathbf{THC} + 5.171 * 10^{-3}\ \mathbf{CBDA} + 8.740 * 10^{-5}\ \mathbf{THCA^2} + 2.699$	0.194	0.602	0.119
4	$\mathbf{Log}(\mathbf{EC_{50}}) = 5.722 * 10^{-3}\ \mathbf{CBD} - 6.423 * 10^{-3}\ \mathbf{THC} + 1.133 * 10^{-4}\ \mathbf{CBDA^2} + 8.788 * 10^{-5}\ \mathbf{THCA^2} + 2.698$	0.202	0.599	0.134
5	$\mathbf{Log}(\mathbf{EC_{50}}) = 6.157 * 10^{-3}\ \mathbf{CBD} - 8.791 * 10^{-5}\ \mathbf{THC^2} + 5.594 * 10^{-3}\ \mathbf{CBDA} + 6.609 * 10^{-3}\ \mathbf{THCA} + 2.615$	0.187	0.605	0.105
6	$\mathbf{Log}(\mathbf{EC_{50}}) = 6.225 * 10^{-3}\ \mathbf{CBD} - 8.889 * 10^{-5}\ \mathbf{THC^2} + 1.233 * 10^{-4}\ \mathbf{CBDA^2} + 6.683 * 10^{-3}\ \mathbf{THCA} + 2.613$	0.196	0.601	0.122
7	$\mathbf{Log}(\mathbf{EC_{50}}) = 6.438 * 10^{-3}\ \mathbf{CBD} - 9.193 * 10^{-5}\ \mathbf{THC^2} + 5.850 * 10^{-3}\ \mathbf{CBDA} + 9.888 * 10^{-5}\ \mathbf{THCA^2} + 2.631$	0.183	0.606	0.096
8	$\mathbf{Log}(\mathbf{EC_{50}}) = 6.489 * 10^{-3}\ \mathbf{CBD} - 9.265 * 10^{-5}\ \mathbf{THC^2} + 1.285 * 10^{-4}\ \mathbf{CBDA^2} + 9.966 * 10^{-5}\ \mathbf{THCA^2} + 2.629$	0.192	0.603	0.113
9	$\mathbf{Log}(\mathbf{EC_{50}}) = 9.093 * 10^{-5}\ \mathbf{CBD^2} - 5.939 * 10^{-3}\ \mathbf{THC} + 4.807 * 10^{-3}\ \mathbf{CBDA} + 5.680 * 10^{-3}\ \mathbf{THCA} + 2.692$	0.183	0.606	0.129
10	$\mathbf{Log}(\mathbf{EC_{50}}) = 9.175 * 10^{-5}\ \mathbf{CBD^2} - 5.993 * 10^{-3}\ \mathbf{THC} + 1.057 * 10^{-4}\ \mathbf{CBDA^2} + 5.730 * 10^{-3}\ \mathbf{THCA} + 2.691$	0.191	0.603	0.144
11	$\mathbf{Log}(\mathbf{EC_{50}}) = 9.552 * 10^{-5}\ \mathbf{CBD^2} - 6.239 * 10^{-3}\ \mathbf{THC} + 5.050 * 10^{-3}\ \mathbf{CBDA} + 8.536 * 10^{-5}\ \mathbf{THCA^2} + 2.709$	0.181	0.607	0.124
12	$\mathbf{Log}(\mathbf{EC_{50}}) = 9.610 * 10^{-5}\ \mathbf{CBD^2} - 6.277 * 10^{-3}\ \mathbf{THC} + 1.107 * 10^{-4}\ \mathbf{CBDA^2} + 8.588 * 10^{-5}\ \mathbf{THCA^2} + 2.708$	0.189	0.604	0.139
13	$\mathbf{Log}(\mathbf{EC_{50}}) = 1.026 * 10^{-4}\ \mathbf{CBD^2} - 8.525 * 10^{-5}\ \mathbf{THC^2} + 5.425 * 10^{-3}\ \mathbf{CBDA} + 6.409 * 10^{-3}\ \mathbf{THCA} + 2.628$	0.172	0.610	0.109
14	$\mathbf{Log}(\mathbf{EC_{50}}) = 1.039 * 10^{-4}\ \mathbf{CBD^2} - 8.629 * 10^{-5}\ \mathbf{THC^2} + 1.197 * 10^{-4}\ \mathbf{CBDA^2} + 6.487 * 10^{-3}\ \mathbf{THCA} + 2.627$	0.182	0.606	0.126
15	$\mathbf{Log}(\mathbf{EC_{50}}) = 1.078 * 10^{-4}\ \mathbf{CBD^2} - 8.954 * 10^{-5}\ \mathbf{THC^2} + 5.698 * 10^{-3}\ \mathbf{CBDA} + 9.631 * 10^{-5}\ \mathbf{THCA^2} + 2.644$	0.169	0.611	0.102

16	$\boldsymbol{Log}(\boldsymbol{EC_{50}}) = 1.087 * 10^{-4}\, \boldsymbol{CBD^2} - 9.032 * 10^{-5}\, \boldsymbol{THC^2} + 1.253 * 10^{-4}\, \boldsymbol{CBDA^2} + 9.716 * 10^{-5}\, \boldsymbol{THCA^2} + 2.643$	0.178	0.608	0.119

Figure S1. Dose-response curves for (A) CBD and CBDA, and (C) Δ^9-THC and Δ^9-THCA [Ref. 15] as agonists at CB_1 receptor, using CP-55940 as a reference compound. Panels (B) and (D) show the dose-response curves for CBD and CBDA, and Δ^9-THC and Δ^9-THCA as antagonists, respectively, with SR-141716 as a reference compound at CB_1 receptor.

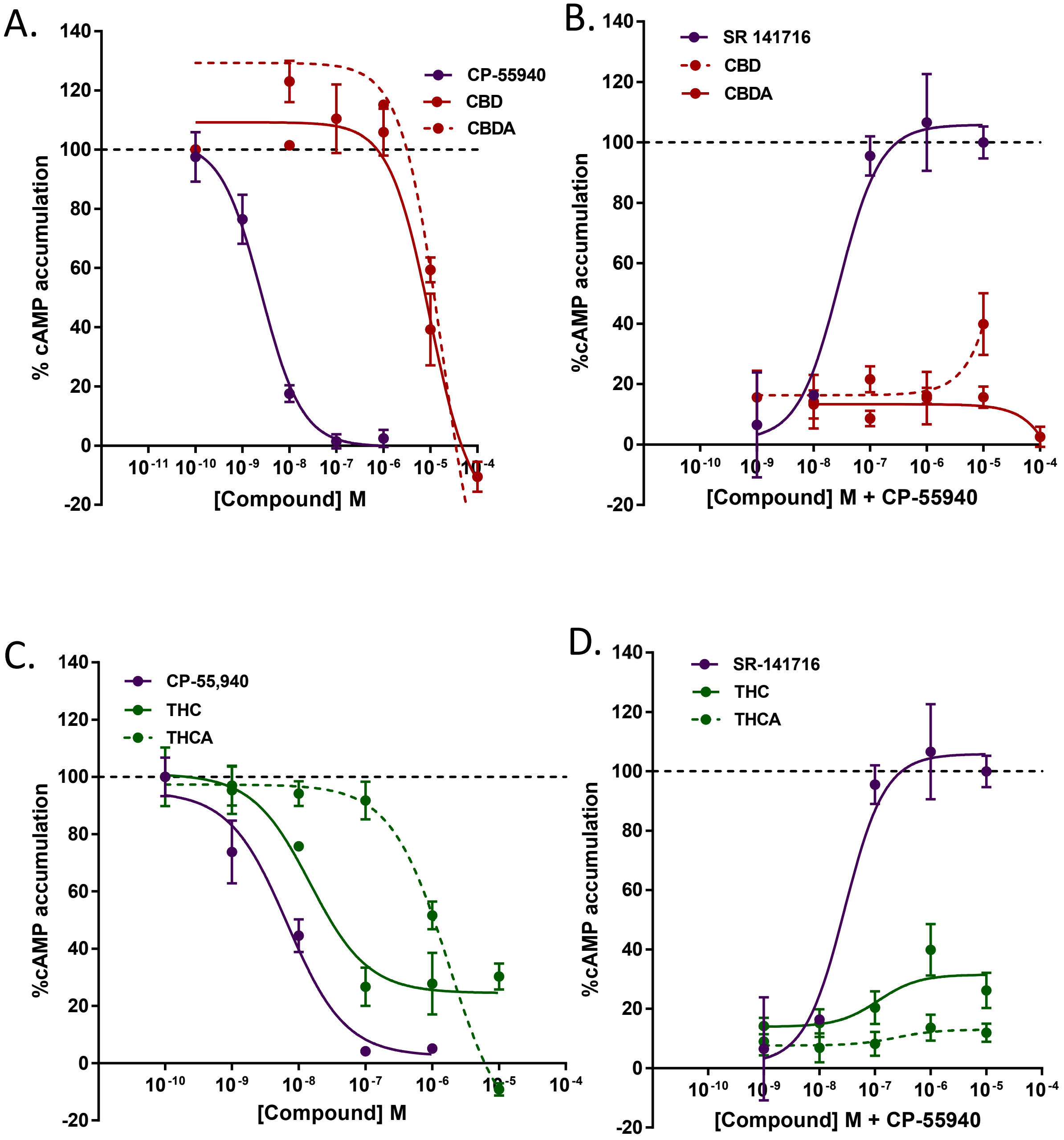

Figure S2. Dose-response curves for (A) CBD and CBDA, and (C) Δ^9-THC and Δ^9-THCA as agonists at CB_2 receptor, using CP-55940 as a reference compound. Panels (B) [Ref. 15] and (D) show the dose-response curves for CBD and CBDA, and Δ^9-THC and Δ^9-THCA as antagonists, respectively, with AM630 as a reference compound at CB_2 receptor.

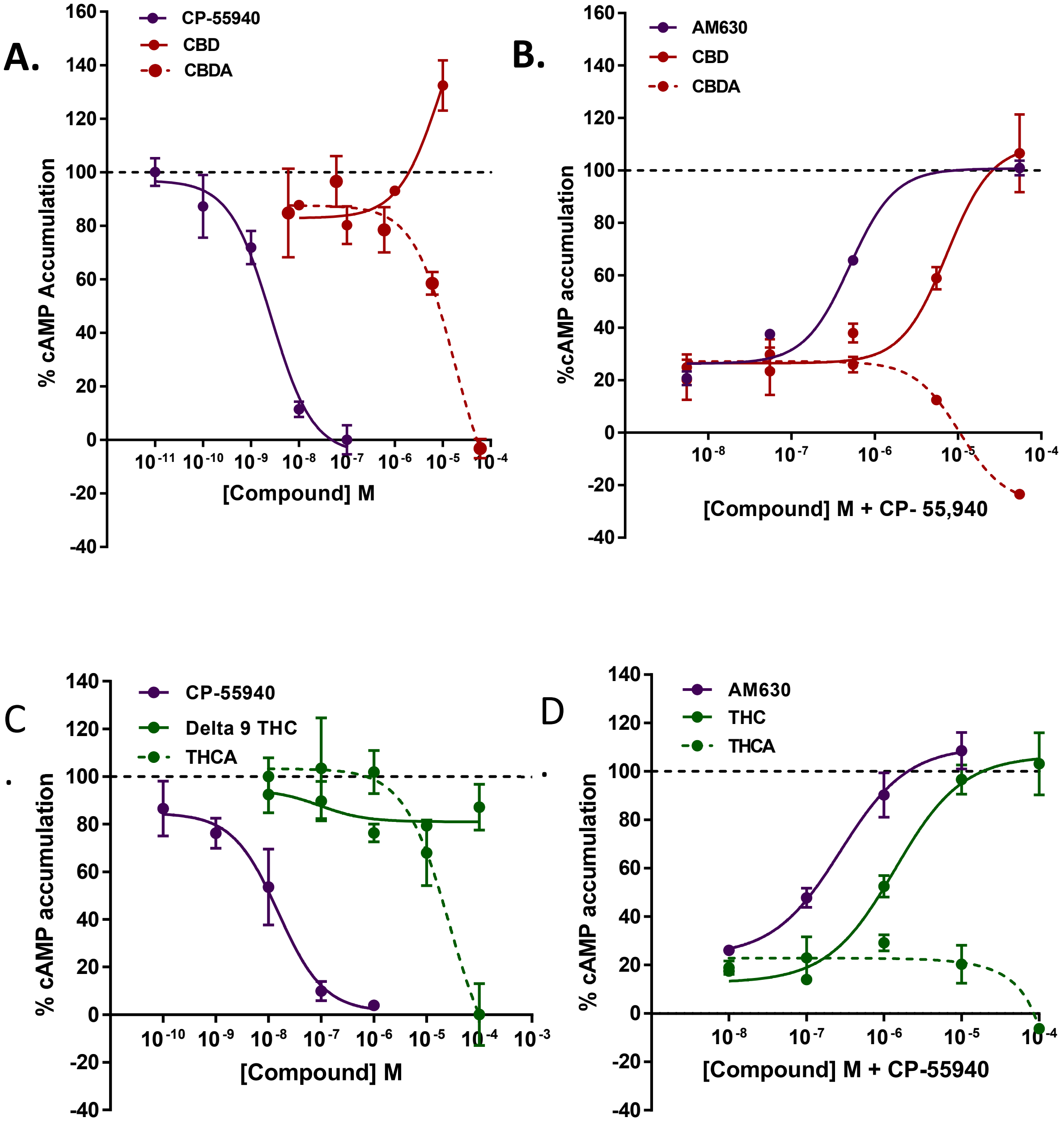

Supplement: Supplementary file 1 [file molecules-25-03466-s001.pdf]
